# Antioxidant activity and interactions between whey protein and polysaccharides from different parts of *Houttuynia cordata*

**DOI:** 10.3389/fnut.2023.1020328

**Published:** 2023-01-25

**Authors:** Xiaocui Liu, Jin Tian, Zhiran Zhou, Yinzhen Pan, Zhongqiao Li

**Affiliations:** College of Food and Bioengineering, Xihua University, Chengdu, China

**Keywords:** *Houttuynia cordata*, polysaccharide-whey protein complex, antioxidant activity, interaction, structural characterization

## Abstract

*Houttuynia cordata* polysaccharides (PSY) are known to exhibit a variety of beneficial activities, but these are currently not specifically utilized in food. Hence, using the two edible parts of *Houttuynia cordata*, a herbaceous plant native to Southeast Asia, this study developed polysaccharides of a stem (HCPS)-whey protein concentrate (WPC) complex and a leaf (HCPL)-WPC complex, and studied their stability, structure and antioxidant activity. The results showed that stability differed in complexes with different proportions, exhibiting only relative stability in the two complexes in which the ratio of HCPS-WPC and HCPL-WPC was 1:4, but increased stability in the HCPL-WPC complex (ζ-potential of HCPL-WPC: | -21.87 mv| >ζ-potential of HCPS-WPC: | -21.70 mv|). Structural characterization showed that there was electrostatic interaction between HCPS and WPC and between HCPL and WPC. The HCPL-WPC was found to have better antioxidant activity. The findings of this study, thus, provide a reference for the development of *Houttuynia cordata* polysaccharide applications in food.

## 1. Introduction

*Houttuynia cordata* is a characteristic, nutritionally rich food in Southwest China, with many Chinese people also utilizing it as a traditional medicine in the treatment of cough, dyspnea, and pneumonia (1). Research has shown that *H. cordata* is rich in phenols, flavonoids, volatile oils, polysaccharides (PSY), and other active substances, *Houttuynia cordata* contains 15.73∼20.29% polysaccharide (2). Shi et al. (3), for example, found that orally administered *H. cordata* PSY could directly regulate the dynamic balance of Th17/Treg cells in the intestines and lung, and reduce lung injury in influenza infected mice. Because of their unique physical and chemical properties and potential biological activity, PSY are often added to food to improve its functional characteristics (4). As yet, few studies have been published on the interaction between *H. cordata* PSY and whey protein (WP), however, there are reports on the interaction between PSY extracted from other plants and WP. Niu et al. (5) studied the interaction mechanism between plantain PSY and WP by preparing a mixture of PSY and WP. At the same time, by comparing the bile acid binding ability of PSY and the PSY-WP mixture, the authors ascertained that the mixture had a threefold greater bile acid binding ability than PSY alone, making it suitable for development into a nutritional additive for the prevention of hyperlipidemia.

Proteins and PSY are two important and abundant biological macromolecules. Commonly, they are separately added to food as functional components (6), to improve the taste, structure, tissue state, stability, apparent characteristics and shelf life of food, while simultaneously increasing its functional characteristics (7). WP is a high-quality natural protein with a high biological titer in milk, in which it accounts for approximately 20% of total protein (8). WP contains β-lactoglobulin, α-lactalbumin, lactoferrin and lysozyme growth factor (9), all trace components that provide a variety of biological activities and health functions. Consequently, WP has the characteristics of high nutritional value, reasonable amino acid composition, easy digestion and absorption by the body, and a high utilization rate of functional activity. It has been found that polysaccharide and WP form complexes through non-covalent and covalent interactions (10). Non-covalent interactions mainly include electrostatic interactions, hydrogen bond actions, hydrophobic interactions and steric exclusion (11); Covalent interactions is mainly the chemical reaction between protein and polysaccharide, such as enzyme cross-linking and non-enzyme cross-linking (12). The interaction between PSY and WP has been widely studied (13) in order to overcome the shortcomings of WP sensitivity to temperature, pH value and ionic strength and, thereby, improve the utilization of WP. For example, WP and PSY were used to prepare covalent complexes through the Maillard reaction, which effectively improved and enhanced the functional properties of WP (14). In addition to the Maillard reaction, the soluble complex formed by the interaction between polysaccharide and WP in an aqueous solution can also be used as a fat substitute, thickener, stabilizer or transport carrier in functional food (15), which not only changes the structure of the food, but also improves its functional characteristics (16).

The edible parts of *H. cordata* include mainly its stems and leaves. In this study, the complex of WPC-HCPS and WPC-HCPL were prepared, the main objective of which was to investigate the effects of proportion on the interaction of the complex.

## 2. Materials and methods

### 2.1. Materials and chemicals

Whey protein concentrate (WPC, 80% protein) was obtained from Yuanye Bio-Technology Co., Ltd. (Shanghai, China). *H. cordata* was purchased from Wuxi Xiaogu E-commerce Co., Ltd. (Chongqing, China). Hydrochloric acid, sodium hydroxide and absolute ethanol were provided by Chengdu Kelong Chemical Co., Ltd. (Chengdu, China). All chemicals were of analytical grade.

### 2.2. Extraction of *H. cordata* stems and leaves (HCPS and HCPL)

The fresh *H. cordata* were dried at 55°C and then the stems (HCPS, 1 kg) and leaves (HCPL, 1 kg) powdered separately and soaked in absolute ethanol overnight at room temperature. Following suction filtration, the HCPS were extracted with deionized water (30 L) at 90°C for 5 h, while the HCPL were extracted with deionized water (50 L) at 90°C for 3 h. The extracts were then concentrated and absolute ethanol was added to the concentrates, whereafter they were left to stand overnight at 4°C, the precipitation then redissolved in deionized water and protein was removed using Sevage reagent. Thereafter, the samples were dialyzed against flowing deionized water for 36 h. The HCPS and HCPL were collected for simple purification by dynamic elution with macroporous resin, then concentrated and vacuum dried. HCPS was mainly composed of Man (4.22%), Rha (24.75%), GlcA (3.67%), GalA (10.42%), Glc (30.42%), and Xyl (20.55%), and HCPL was mainly composed of Man (1.33%), Rha (3.40%), GlcA (1.50%), GalA (2.19%), Glc (8.30%), Xyl (4.12%), Gal (1.50%), and Ara (61.33%) by monosaccharide analysis (17).

### 2.3. Preparation of complexes

Whey protein concentrate stock solution (10% w/v) and HCPS and HCPL stock solutions (0.25% w/v) were prepared in deionized water and stored at 4°C to ensure both protein dissolution and polysaccharide hydration. The WPC stock solution and HCPS and HCPL stock solutions were mixed separately at volume ratios of 1:0, 4:1, 3:2, 1:1, 2:3, 1:4, and 0:1. The pH value of each mixed solution was adjusted from 6.77 to 7, and the WPC-HCPS and WPC-HCPL solutions were kept overnight at 4°C, then restored to room temperature before use.

### 2.4. Turbidimetric analysis

The turbidimetry of the mixed solutions at different volume ratios was measured at 600 nm using a Fluostar Omega microplate reader (SpectraMax i3x, Molecular Devices, USAx), using the method described by Aryee and Nickerson (18). The mixed solutions were diluted 1:10. Deionized water was used as the control.

### 2.5. Particle size and zeta potential measurements

The laser particle size analyzer (Zetasizer Nano ZEN3600, Malvern, UK) was used to determine the particle size and zeta potential of the two mixed solutions (WPC-HCPS and WPC-HCPL) with different proportions. The average particle size was obtained *via* equilibrium for 2 min at 25°C. The zeta (ζ)-potentials of the complexes (WPC-HCPS and WPC-HCPL) were measured and the ζ-potential was estimated according to the Formula 1:


(1) $$U_{E}=\frac{2\text{ε }\times \;\zeta \;\times \;f\left(\kappa \,\alpha \right)}{3\text{η}}$$

where U_*E*_ is conductivity mobility, ε is the permittivity [F(Farad/m)], η is the solution viscosity (mPa.s), κ is the Debye length (nm^–1^), α is the particle radius (nm), and f(κα) = 1.5.

### 2.6. Rheological measurement

A rheometer (MCR302, Anton paar, Austria) was used to measure the mixed solutions in different proportions, and a 50 mm aluminum plate fixture was selected for the measurement. The distance between the fixture and the Peltier plate was 1 mm. Measurements were carried out at 25°C with a shear rate in the range of 0.1–1,000 s^–1^.

### 2.7. Differential thermogravimetric measurements

The differential thermogravimetric (DTG) curves of the mixed solutions at different volume ratios were recorded using a DTG-60 analyzer (Shimadzu, Japan). The scanning was determined in the range of 30–800°C, the heating rate was 10°C/min, and the nitrogen flow rate was 30 mL/min.

### 2.8. Ultraviolet spectrophotometric determination

Each sample was placed in a quartz colorimetric cell for individual ultraviolet (UV) spectrophotometric determination, in which the absorption spectra were scanned in the wavelength range of 230–500 nm.

### 2.9. Fourier transform infrared spectroscopy

The Fourier transform infrared (FTIR) spectra of lyophilized WPC-HCPS and WPC-HCPL were recorded using an FTIR spectrophotometer (Spectrum Two N, PerkinElmer, USA) within a range of 400–4,000 cm^–1^ *via* 32 scans at a resolution of 4 cm^–1^. The lyophilized samples were mixed well with potassium bromide and then compressed, after which each milligram of sample was further mixed with 100 milligrams of potassium bromide.

### 2.10. Scanning electron microscopy

Complex solutions were freeze-dried and then observed *via* a scanning electron microscope (SEM; Gemini 300, ZEISS, Germany), according to the method of Timilsena et al. (19). The lyophilized samples were plated with gold and also then studied *via* SEM with a scanning voltage of 3 kV.

### 2.11. Antioxidant activity

#### 2.11.1. Scavenging efficiency of DPPH ⋅

The scavenging efficiency of complexes for DPPH ⋅ were determined using the methods of Cheng et al. (20). With vitamin C as the control, 2 mL of each complex was mixed with 0.1 mM DPPH ⋅ solution of the same volume and then shaken well. The system was then left to react in a dark place for 30 min, whereafter it was measured at 510 nm. The sample and the DPPH ⋅ solution were then replaced with deionized water and absolute ethanol, respectively. The scavenging efficiency was then calculated using Formula 2:


(2) $$\mathrm{Scavenging}\mathrm{efficiency}\left(\%\right)=\frac{{\text{A}}_{0}-\left({\text{A}}_{1}-{\text{A}}_{2}\right)}{{\text{A}}_{0}}\times 100$$

where A_0_ is deionized water and DPPH ⋅ solution, A_1_ is complex and DPPH ⋅ solution, A_2_ is complex and absolute ethanol.

#### 2.11.2. Scavenging efficiency of ⋅OH

The scavenging efficiency of ⋅OH was assessed, with reference to the method described by Tian et al. (21). The two complexes were mixed well with a ferrous sulfate (FeSO_4_) solution (9 mmol/L) and a hydrogen peroxide (H_2_O_2_) solution (6 mmol/L) under a constant temperature 37°C for 10 min, whereafter a salicylic acid solution (9 mmol/L) was added to this mixture and then left to react at 37°C in the dark for 30 min. Finally, the mixture was measured at 600 nm. The scavenging efficiency was then calculated using Formula 2:


(3) $$\mathrm{Scavenging}\mathrm{efficiency}\left(\%\right)=\frac{{\text{A}}_{0}-\left({\text{A}}_{1}-{\text{A}}_{2}\right)}{{\text{A}}_{0}}\times 100$$

where A_0_ is deionized water+ FeSO_4_+H_2_O_2_+ salicylic acid solution, A_1_ is complex+FeSO_4_+H_2_O_2_+salicylic acid solution, and A_2_ is complex+ FeSO_4_+ deionized water+ salicylic acid solution.

#### 2.11.3. Scavenging efficiency of ABTS^+^⋅

The scavenging ability of ABTS^+^⋅ was determined according to the method described by de Falco et al. (22). ABTS and potassium persulfate were mixed and stored at 4°C. The ABTS^+^⋅ stock solution was then diluted to an OD value of 0.7–0.8 before use. The samples were mixed with the ABTS^+^⋅ solution, left to react for 3 min and then measured at 734 nm. Deionized water was used as the blank control. The calculation formula was as follows:


(4) $$\mathrm{Scavenging}\mathrm{efficiency}\left(\%\right)=\frac{{\text{OD}}_{\text{control}}-{\text{OD}}_{\text{sample}}}{{\text{OD}}_{\text{control}}}\times 100$$

Where, OD_*control*_ is the blank control and OD_*sample*_ is the sample.

#### 2.11.4. Reduction capacity of Fe^3+^

The reduction ability of the sample to Fe^3+^ was determined using the method reported by Yildirim et al. (23). The samples were mixed well with phosphate buffer (0.2 mol/L, pH 6.6) and potassium ferricyanide solution (1%), then left at 50°C for 20 min, and then cooled. Trichloroacetic acid (10%) was added to the above mixture. After being left to stand for 10 min, the absorbance of the mixture was measured at 700 nm. The reduction capacity was expressed in terms of OD value.

### 2.12. Statistical analysis

All the results were expressed as the mean ± standard error (SE) in the tables. Differences between groups were determined *via* Tukey’s test analysis using SPSS 26 statistics software. A probability value of *p* less than 0.05 was considered significant.

## 3. Results and discussion

### 3.1. Turbidimetric and particle size analysis

**FIGURE 1:**
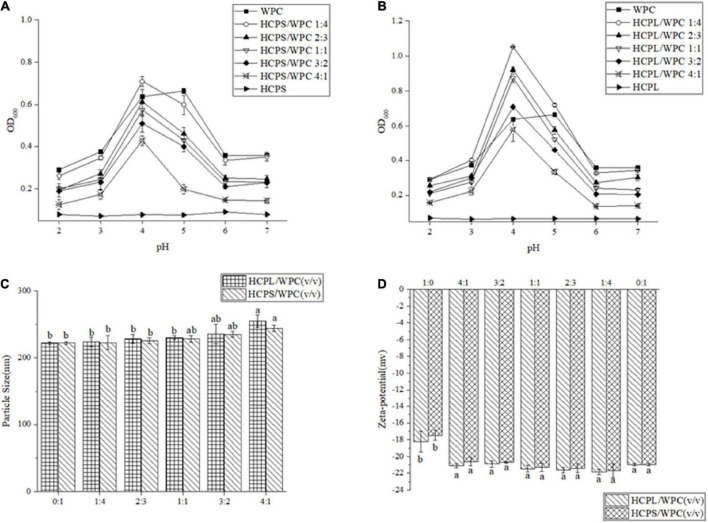
Curves of the turbidity of HCPS-WPC **(A)** and HCPL-WPC **(B)** at different volume ratios to the pH. Particle size **(C)** of complexes. The ζ–potential of complexes **(D)**.

Turbidity value can reportedly show the formation of a complex or condensate during the interaction between polysaccharide and protein (24). In this study, the OD of the HCPS and HCPL changed little, indicating that they were not affected by acidification in Figure 1 (25). Maximum OD was reached at approximately pH 5.0 due to the isoelectric point (pI) of the WPC in the range of pH 5.0–5.2 (Figure 1) (13). The turbidity curve of the WPC first increased and then decreased. The OD of both the HCPS-WPC and HCPL-WPC peaked at pH 4.0 and the curve shapes of the complexes were similar to that of the WPC. The same phenomena were previously observed in WP isolate and a carrageenan system (26). Compared with the WPC, the peak of complexes shifted to a lower pH, thus demonstrating the electrostatic interaction between HCPS or HCPL and WPC, as shown in Figure 1 (27). The HCPS-WPC complexes were observed with the peak of turbidity at a ratio of 1:4 (pH 4.0). At this time, the complexes contained a significantly large amount of coacervates (Figure 1A). The HCPL-WPC complexes were also at the maximum OD at pH 4.0 when the ratio was 1:4 (Figure 1B). However, the OD of HCPL-WPC complexes were higher than that of the HCPS-WPC at the same ratio (pH 3.0–5.0). This phenomenon can be explained by the precipitate yield of the HCPS-WPC complexes, which was less than that of the HCPL-WPC (28). Overall, 1:4 was found to be the optimal ratio for the interaction between HCPS or HCPL, and WPC. Further understanding of the interactions between HCPS and WPC, and HCPL and WPC, under neutral pH conditions could improve the application of HCPS and HCPL in the field of functional food. Therefore, this paper studied the effect of protein-polysaccharide ratio on the type and degree of interaction between HCPS or HCPL and WPC at pH 7.

As shown in Figure 1C, particle sizes were found to increase with the increase in HCPS and HCPL. Furthermore, the particle size of the HPCL-WPC was greater than those of the HPCS-WPC. The maximum difference between the particle size of the WPC and that of the complexes was only 32.6 nm, which could be related to the relatively dispersible structure of complexes (29).

### 3.2. ζ–potential

The ζ-potential of the HCPS-WPC and HCPL-WPC complexes could reflect the interaction between polysaccharide and WP, as well as their stability. A high absolute value of ζ-potential is indicative of a stable system (30). The ζ-potential of HCPS-WPC and HCPL-WPC is shown in Figure 1D. HCPS, HCPL and WPC were all found to have negative ζ-potential at pH 7.0, and the ζ-potentials of complexes with different volume ratios were insignificant. However, ζ-potential was below that of the WPC when the HCPL-WPC was at a volume ratio of 3:2 and in the HCPS-WPC at ratios of 3:2 and 4:1, indicating that the negative charges of HCPS and HCPL interacted with positive charges of WPC (31). As shown in Figure 1D, the highest ζ-potential was observed in both HCPS-WPC (-21.70 ± 0.85 mv) and HCPL-WPC (-21.87 ± 0.29 mv) at ratios of 1:4, because the increase of WPC led to the increase in ζ-potential. Moreover, the potential value of the HCPL-WPC was comparatively higher than that of the HCPS-WPC in the same ratio.

### 3.3. Thermogravimetric analysis

**FIGURE 2:**
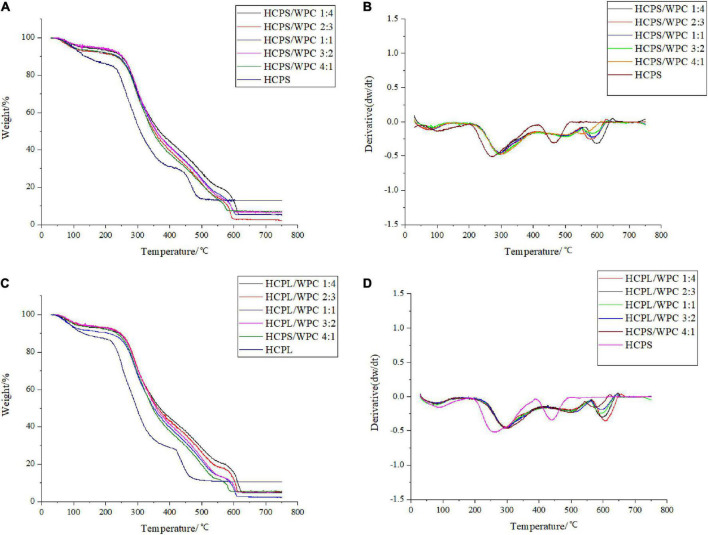
Thermogravimetry curve of HCPS-WPC **(A)** and HCPL-WPC **(C)**. The differential thermogravimetric curve of HCPS-WPC **(B)** and HCPL-WPC **(D)**.

Thermogravimetric analysis revealed the thermal degradation of both HCPS-WPC and HCPL-WPC, as shown in Figure 2. With the gradual increase in program temperature, thermogravimetric change occurred in roughly three stages. For HCPS-WPC, the first stage of weightlessness occurred mainly in the range of 40–120°C (Figure 2A), and was due to the water in the sample. Concurrently, the derivative thermogravimetric (DTG) curve appeared to peak at the corresponding temperature (Figure 2B). Subsequently, the second stage of weightlessness was seen in the range of 220–400°C (Figure 2A). The thermogravimetric loss of approximately 50% in this stage was attributed to structural water and the cleavage of protein and polysaccharide (32). The thermal decomposition rate of the HCPS-WPC shifted to a high temperature and increased by 30°C compared with the HCPS, thus indicating that the HCPS-WPC had better thermal stability (Figure 2B). Finally, carbonization occurred in the third stage (530–650°C), in which the average loss rate was 22% (33), and the carbonization temperature of the HCPS-WPC was higher than that of the HCPS.

For the HCPL-WPC, thermogravimetric analysis showed similar results to the thermogravimetric changes occurring in the HCPS-WPC. However, the thermal decomposition temperature of the HCPL-WPC was 40°C higher than that of HCPL in the second weightlessness stage, and the weight loss temperature in the third stage was 540–660°C. In brief, the interaction of WPC with HCPS and HCPL improved the thermal stability of the complexes, which was also previously observed in a mixture of oat β-glucan/soy protein isolates (34).

### 3.4. Rheological analysis

**FIGURE 3:**
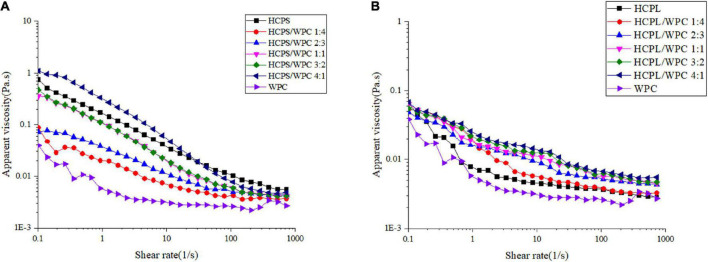
The viscosity curve of HCPS-WPC **(A)** and HCPL-WPC **(B)**.

The apparent viscosity changes in the HCPS-WPC complexes at different volume ratios were selected for study (Figure 3A). Shear thinning was observed in all complexes, in which viscosity decreased with the increase of shear rate. The viscosity was dependent on the volume ratio in the HCPS-WPC, with a declining trend of apparent viscosity in line with the increasing WPC. The apparent viscosity of the HCPL-WPC (Figure 3B) also decreased with the increase of shear rate, and the viscosity difference between the HCPL-WPC complexes was not significant. These complexes exhibited the characteristics of pseudoplastic fluids (35). However, the viscosity of the HCPS-WPC was higher than that of the HCPL-WPC, possibly due to the higher pectin content of HCPS.

### 3.5. UV analysis

**FIGURE 4:**
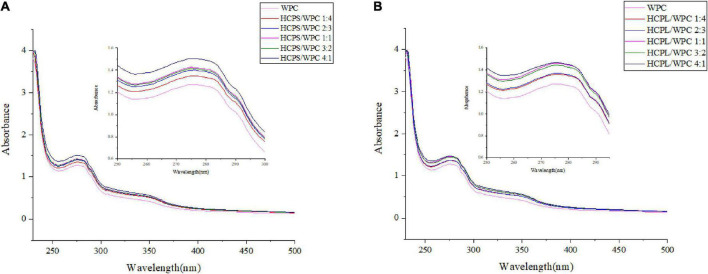
The ultraviolet-visible spectroscopy of HCPS-WPC **(A)** and HCPL-WPC **(B)**.

The interactions between HCPS and WPC, and HCPL and WPC, were further explored *via* UV-vis spectroscopy. An absorption peak of amino acid residues was observed at 260–280 nm. Changes in peak intensity could be used to indicate the strength of an interaction (36). As shown in Figure 4, the WPC had a maximum absorption peak at 270–280 nm, corresponding to the vibration of Tyr (277 nm). After WPC was mixed with HCPS and HCPL, respectively, the absorption peak increased significantly, which might have been due to the formation of covalent conjugated complexes (37). More importantly, the peak change in the HCPL-WPC was greater than that of the HCPS-WPC, indicating that the HCPL-WPC interaction was stronger. As is evident in Figure 4, the peak positions of the complexes changed and gradually increased with the increase in PSY, possibly due to the microenvironmental change of amino acids. Thus, these results showed that the structure and microenvironment of the amino acid residues in WPC might be affected by changes in the polysaccharide-protein ratio which, in turn, affects the interactions between HCPS or HCPL and WPC.

### 3.6. FTIR analysis

**FIGURE 5:**
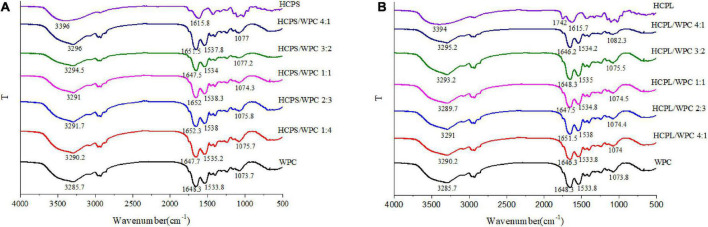
Fourier transform infrared spectra of the HCPS-WPC **(A)** and HCPL-WPC **(B)**.

The FTIR spectroscopy in this study provided more information about the interaction between PSY and WPC, as shown in Figure 5. The spectrum of HCPS revealed that the broad band at 3,396 cm^–^1 was attributed to O–H vibration, while the stretching vibration of C = O on carbonyl groups was observed at 1615.8 cm^–1^ (Figure 5A). For WP, a 1648.3 cm^–1^ band was derived from the C = O stretching of amide I, while the C–N stretching of the amide II peak was observed at 1533.8 cm^–1^, and the strong band at 3285.7 cm^–1^ was associated with O–H stretching vibration. In the HCPS-WPC complexes, with the increase of WPC and the decrease of HCPS, the O-H absorption peak shifted, it can be inferred the presence of hydrogen bonding and electrostatic interactions between polysaccharide and WP (38). Meanwhile, the amide I peaks of the HCPS-WPC complexes shifted and the peaks related to the amide II were shifted to the higher wavenumber. The shifting of amide characteristic peaks was due to the effect of HCPS-WPC electrostatic interactions on the α -helix structure of the WPC (Figure 5A) (39). Furthermore, compared to the native HCPS, the C = O on carboxyl groups was not observed in the HCPS-WPC complexes, and the bands ranged from 1074.3 to 1077.2 cm^–1^.

The HCPL and HCPL-WPC complexes are depicted in Figure 5B. In the HCPL, the 3,394 cm^–1^ band was attributed to the O–H stretching vibration, while the distinctive bands at 1,742 and 1,615 cm^–1^ were related to the stretching vibration of C = O on the carboxyl and carbonyl groups, respectively. The spectrum of HCPL-WPC complexes showed two important bands in the ranges of 1646.2–1651.5 cm^–1^ and 1533.8–1,538 cm^–1^, associated with the shifting of amide I and amide II, respectively. One samples band range from 1074 to 1082.3 cm^–1^ was also observed in the HCPL-WPC complexes (35).

In general, the secondary structure and groups of the WPC were affected by additions of HCPS and HCPL. The contribution of the electrostatic interactions between the carbonyl groups of HCPS or HCPL and the amino groups could explain the shift in amide peaks. Similar interactions have been previously reported in the complex coacervation of pea protein isolate and tragacanth gum (30).

### 3.7. SEM analysis

**FIGURE 6:**
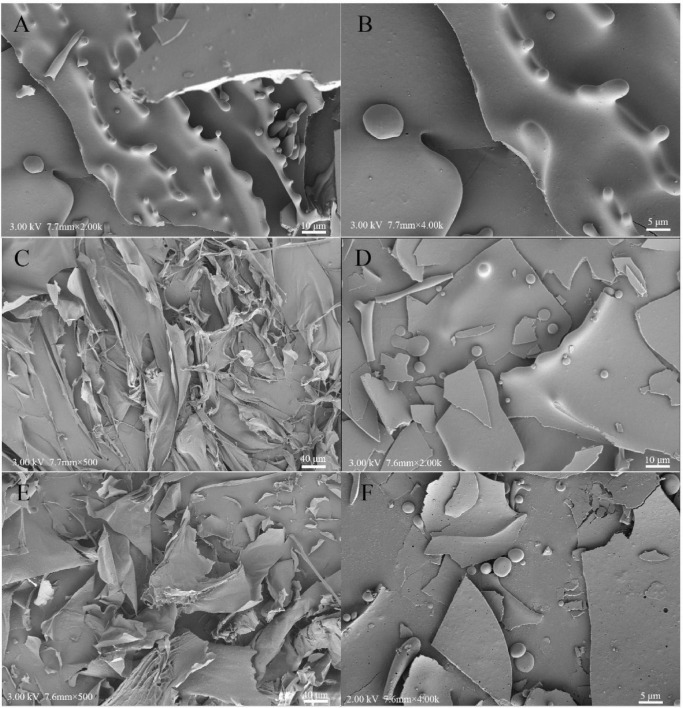
Scanning electron microscope observations of WPC **(A,B),** HCPS **(C)**, HCPL **(E)**, HCPS-WPC 1:4 **(D)** and HCPL-WPC 1:4 **(F)**.

The morphological microstructures of the WPC, HCPS, HCPL, HCPS-WPC, and HCPL-WPC complexes at the ratio of 1:4 are presented in Figure 6. The microstructure of pure WPC was found to be orderly and complete, while its internal structure was mainly spherical (Figures 6A, B). The electron microscope image showed that the surface of HCPS was smooth and intertwined, and the structure was irregular (Figure 6C). HCPL was found to have a more dispersed and flakey structure, indicating it to be mainly amorphous (Figure 6E). In the microstructure of HCPS-WPC (Figure 6D), a smooth and blocky surface was observed, and WPC was adsorbed concurrently by the HCPS. The microstructure of HCPL-WPC was found to be similar to that of the HCPS-WPC, with WPC similarly adsorbed by the HCPL. In brief, the interactions between HCPS or HCPL and WPC were evident. The smooth surface of the complex microstructures could have been due to the use of metal coating (40).

### 3.8. Antioxidant activity

**FIGURE 7:**
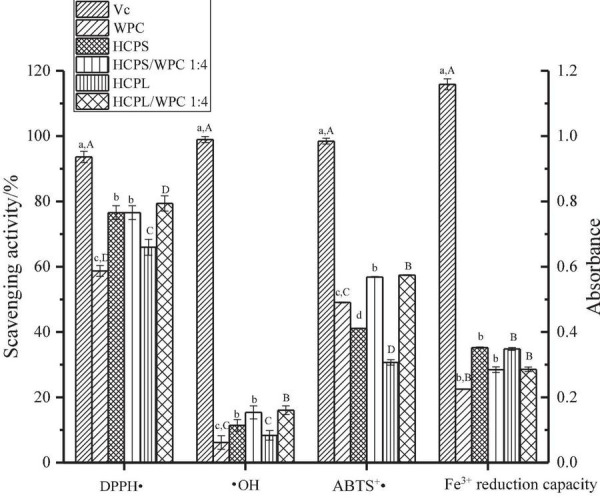
The antioxidant capacity analysis. Different lowercase letters indicate significant differences in HCPS-WPC (*p* < 0.05). Different uppercase letters indicate significant differences in HCPL-WPC (*p* < 0.05).

The antioxidant capacities of the complexes HCPS, HCPL and WPC are shown in Figure 7. The scavenging capacities of the DPPH ⋅, ⋅ OH and ABTS^+^⋅ of the complexes were higher than those of the pure HCPS, HCPL, and WPC, indicating the synergistic effect between HCPS or HCPL and WPC. Among them, the antioxidant capacity of the HCPL-WPC was slightly higher than that of the HCPS-WPC, but the antioxidant capacity of the HCPL was lower than that of the HCPS, which might have been due to the stronger interaction and synergy between HCPL and WPC. These findings concur with the results of the UV analysis in this study. However, the Fe^3+^ reduction capacity of the complexes was lower than those of the HCPS and HCPL, it might be that the interaction between WPC and polysaccharide would inhibit the Fe^3+^ reduction capacity of complexes. In general, the antioxidant activity of the complexes was superior. For better comparison, vitamin C was used as a positive control. Furthermore, in the study of flaxseed gum-WP isolate, the antioxidant capacity of the mixture was also found to be superior to the compounds alone (17).

## 4. Conclusion

This study explored the interactions between HCPS and WPC, and HCPL and WPC. Turbidity analysis revealed that the interactions of the complexes were greatest when pH was 4 and the mixing ratios of HCPS-WPC and HCPL-WPC were 1:4. The physicochemical properties and structural characterization of the two complexes under neutral conditions were also examined and it was found that there was electrostatic interaction between HCPS/HCPL and WPC under neutral conditions, and that the addition of WPC improved the thermal stability of the complexes. Rheological studies showed that HCPS-WPC and HCPL-WPC were pseudoplastic fluids, and FTIR further confirmed the interaction between HCPS/HCPL and WPC. Comparative analysis of the two complexes showed that the HCPL-WPC system was more stable and also had a higher level of antioxidant activity. This research, thus, provides in-depth information regarding the interactions between HCPL and WPC, which can contribute to the development of functional foods with special nutritional value.

## Data availability statement

The raw data supporting the conclusions of this article will be made available by the authors, without undue reservation.

## Author contributions

JT: formal analysis and writing—original draft preparation. XL: methodology, formal analysis, validation, project administration, and funding acquisition. ZZ: validation and data curation. YP: validation and writing—review and editing. ZL: writing and review. All authors read the manuscript, critically examined the important intellectual content, and approved the final version.
